# High-Density Lipoprotein Metabolism and Function in Cardiovascular Diseases: What about Aging and Diet Effects?

**DOI:** 10.3390/nu16050653

**Published:** 2024-02-26

**Authors:** Mojgan Morvaridzadeh, Nada Zoubdane, Javad Heshmati, Mehdi Alami, Hicham Berrougui, Abdelouahed Khalil

**Affiliations:** Department of Medicine, Geriatric Service, Faculty of Medicine and Health Sciences, Université de Sherbrooke, Sherbrooke, QC J1H 4N4, Canada; mojgan.morvaridzadeh@usherbrooke.ca (M.M.); zoubdane.nada@usherbrooke.ca (N.Z.); javad.hishmati@usherbrooke.ca (J.H.); mehdi.alami@usherbrooke.ca (M.A.); hicham.berrougi@usherbrooke.ca (H.B.)

**Keywords:** HDL, CVD, aging, inflammation, oxidative stress, diet, lipoproteins

## Abstract

Cardiovascular diseases (CVDs) have become the leading global cause of mortality, prompting a heightened focus on identifying precise indicators for their assessment and treatment. In this perspective, the plasma levels of HDL have emerged as a pivotal focus, given the demonstrable correlation between plasma levels and cardiovascular events, rendering them a noteworthy biomarker. However, it is crucial to acknowledge that HDLs, while intricate, are not presently a direct therapeutic target, necessitating a more nuanced understanding of their dynamic remodeling throughout their life cycle. HDLs exhibit several anti-atherosclerotic properties that define their functionality. This functionality of HDLs, which is independent of their concentration, may be impaired in certain risk factors for CVD. Moreover, because HDLs are dynamic parameters, in which HDL particles present different atheroprotective properties, it remains difficult to interpret the association between HDL level and CVD risk. Besides the antioxidant and anti-inflammatory activities of HDLs, their capacity to mediate cholesterol efflux, a key metric of HDL functionality, represents the main anti-atherosclerotic property of HDL. In this review, we will discuss the HDL components and HDL structure that may affect their functionality and we will review the mechanism by which HDL mediates cholesterol efflux. We will give a brief examination of the effects of aging and diet on HDL structure and function.

## 1. Introduction

Cardiovascular disease (CVD) continues to be the leading global cause of death, as indicated by the Global Burden of Disease (GBD) study, which reported an estimated 17.8 million CVD-related deaths worldwide in 2017 [1]. Also, in Europe, CVD persists as the predominant cause of death [2]. Strokes and heart attacks appear to be the most prevalent cardiac events documented with about 80% of all recorded cases of mortality [3]. Atherosclerotic cardiovascular disease (ASCVD) stands out as the primary contributor to overall CVD mortality [4]. 

Numerous studies have attributed protective proprieties against CVDs to HDL [5,6]. These studies suggest that low levels of HDL were associated with an increased risk of stroke and heart attack. Moreover, a systematic review and meta-analysis of 23 studies revealed analogous results, establishing an association between diminished levels of HDL and increased risk of coronary artery disease (CAD) in Asian populations [7]. Furthermore, Wilson et al. [6] asserted that low HDL levels increased the risk of mortality. Conversely, some studies failed to corroborate this correlation [8,9], with contradictory findings from Chang et al. [10] suggesting a paradoxical association between high levels of HDL and elevated mortality risk in individuals with CAD. Despite high levels of HDL, there is no assured protection against overall health risks. Unlike Low-Density Lipoprotein (LDL), HDL appears to exclusively correlate with cardiovascular risk in healthy individuals [11]. An observed U-shaped curve in the association between HDL and mortality risk indicates that both concentrations below 30 mg/dL and above 70 mg/dL are linked to increased all-cause mortality risk [12]. One explanation mentioned for the U-shaped link between HDL and cardiovascular risk [12,13] is genetic mutations causing excessively high HDL, associated with adverse cardiovascular risk. Significantly elevated HDL may indicate dysfunctional HDL in some individuals, potentially amplifying cardiovascular risk. Feng et al. proposed that the transfer of free cholesterol to HDL during lipolysis of triglyceride-rich lipoproteins by lipoprotein lipase might contribute to this U-shaped relationship [14]. Moreover, Takaeko et al. [14] demonstrated that exceptionally high levels of HDL were significantly linked to endothelial dysfunction in patients not on lipid-lowering therapy, even after adjusting for traditional cardiovascular risk factors [14]. However, it is important to consider the gender of participants, as a recent study [15] indicated that high HDL levels increased CVD risk in men rather than in women. Nevertheless, because HDLs are a dynamic parameter, in which HDL particles present different atheroprotective properties, it remains difficult to interpret the association between HDL level and CVD risk [16].

HDL is considered the most complex lipoprotein category, comprising several particles that differ in size, structure, composition, and main functions, including antioxidant capacities and anti-inflammatory actions. This lipoprotein’s widely established functions are essential for cardiovascular protection and include the function of reverse cholesterol transport (RCT) [17], inhibition of lipid peroxidation [18], and suppression of inflammation [19]. 

Aging is linked with the deterioration of numerous physiological processes and physical conditions, contributing to an increased risk of various disorders and diseases. Consequently, age is recognized as the primary risk factor for CVDs. In comparison to other health diseases, CVDs convey the largest burden not only for the older population, but also for families and health care systems [20]. From an economic point of view, a recent population-based cost study concluded that the economic cost of CVDs across the 27 European Union countries was about EUR 282 billion annually. Notably, cerebrovascular and coronary heart diseases represented the costliest ailments, with approximately EUR 76 billion and EUR 77 billion, respectively [21]. With the lack of any approved curative therapy, this economic burden is anticipated to escalate alongside the increasing elderly population [22]. The aging process contributes to an augmented prevalence of heart diseases and various direct risk factors for CVDs, including blood pressure, blood lipids, oxidative stress, inflammation, atherosclerosis, metabolic changes, cellular senescence, and endothelial dysfunction [23,24]. Furthermore, in the elderly population, several functions of HDL become impaired by diseases such as diabetes, metabolic syndrome, chronic inflammation, chronic kidney disease, and atherosclerosis [23,25,26]. Taking into account all these factors, aging emerges as a substantial determinant of HDL functions [27]. 

The outcome of studies concerning the relation between HDL particles (small and large) and CVD risk has been contentious [28,29,30]. Conversely, the cholesterol efflux capacity (CEC) of HDL is more practical than HDL level alone in predicting the risk of CVD [31,32]. Another study indicated that, even in comparison to CEC, HDL particle number was a stronger inverse predictor for CVD risk [33]. However, while HDL concentration is commonly used in clinical practice, recent research has suggested that HDL particle number may offer a more accurate prediction of cardiovascular risk [34]. Ultimately, it can be inferred that there has been a heightened effort to identify simpler and more precise indicators for assessing, predicting, or treating CVDs. Although some studies attempted to elucidate the differences in HDL structure/function under normal circumstances compared to a specific disease, inflammation, or oxidative stress situation, there is insufficient research comparing HDL structure/function in the absence or presence of CVD risk factors. Thus, there is a pressing urge to amass this knowledge and comprehend HDL modifications through aging and CVDs. This review endeavors to provide a fresh understanding of HDL structure, metabolism, and function along with the association between HDL and CVD in elderly people. Readers will gain a comprehensive perspective on the role of HDL, particularly in CVD patients, and how aging can affect these mechanisms. 

## 2. HDL Structure

Over the past few decades, there has been increased interest in the structure of HDL. Furthermore, an HDL particle comprises a central core of esterified cholesterol, surrounded by a surface monolayer of phospholipids, free cholesterol, and apolipoproteins [35]. The predominant lipid composition of HDL comprises 35–50% phospholipids, 30–40% cholesterol esters, 5–12% triglycerides, 5–10% sphingolipids, and 5–10% free cholesterol [36]. Comprising more than 200 lipid species, HDL can be categorized into neutral hydrophobic lipids and amphipathic lipids [36]. The former includes esterified cholesterol and triglycerides within the core, while the latter consists of free cholesterol and phospholipids, primarily on the HDL surface. Recent studies revealed that some of the lipids that are carried by HDL can be changed into potent bioactive molecules. For instance, phosphatidylcholine (PC), the most prevalent phospholipid in HDL, is extremely susceptible to oxidation, which after hydrolysis generates the highly reactive and damaging Lysophosphatidylcholine (LPC) [37]. 

Beyond lipids, over 85 protein species are associated with HDL, contributing to various functions including lipid transport and metabolism, hemostasis, immune responses, metal binding, and vitamin transportation. Key apolipoproteins (apo) within HDL include apo A-I, apo A-II, apo A-IV, apo C-I, apo C-II, apo C-III, apo E [38], apo L1, and apo M [39]. Nevertheless, apo A-I and apo A-II are the major proteins that form HDL and serve as the core structural proteins, constituting nearly 70% and approximately 20% of HDL protein size, respectively.

Apo A-Ⅰ is synthesized in the intestine and liver, while apo A-Ⅱ is synthesized in the liver and is present in nearly two-thirds of HDL particles in humans [40]. Apo A-Ⅰ facilitates HDL cholesterol efflux through ATP-binding cassette transporter A1 (ABCA1), a cell membrane protein that transports cholesterol, phospholipids, and other metabolites from cells to lipid-depleted HDL apolipoproteins [41]. Apo A-I and apo C-I activate lecithin–cholesterol acyltransferase (LCAT), which esterifies plasma-free cholesterol. Apo C-II stimulates the activity of plasma lipoprotein lipase (LPL), and is present in chylomicrons, very low-density lipoprotein (VLDL), and intermediate-density lipoproteins (IDLs). Apo C-III exhibits an inhibitory effect toward the LPL [42]. Apo E is a ligand for numerous members of the LDL-receptor (LDLr) family, expressed in the liver, brain, and other tissues. The apo E–LDLr interaction plays an essential role in CVDs, as evidenced by mice that completely lack apo E becoming extremely hypercholesterolemic and developing extensive atherosclerotic lesions [43]. 

HDL particles exhibit wide variations in particle size, composition, charge, and function, categorized by different methods including density gradient ultracentrifugation (HDL2 and HDL3), gradient gel electrophoresis (HDL 2a, 2b, 3a, 3b, and 3c), 2-dimensional gel electrophoresis (pre-beta 1 and 2, and alpha 1, 2, 3, and 4), Lipoprint polyacrylamide electrophoresis gel (HDL_1_ to HDL_10_), and nuclear magnetic resonance (NMR) (large, medium, and small) [44]. 

**Figure 1:**
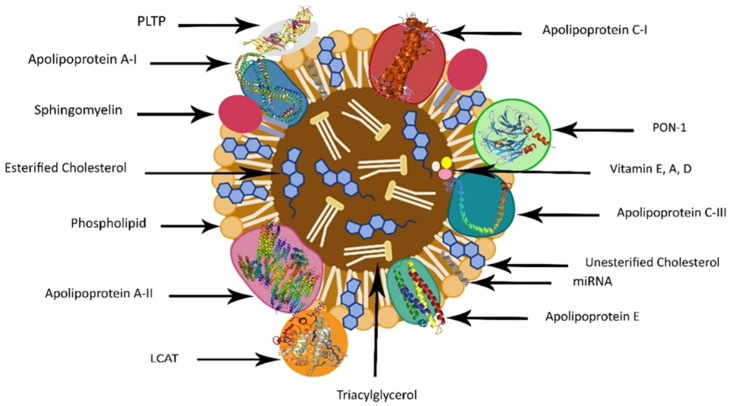
The main structure of a spherical HDL. LCAT: Lecithin–Cholesterol Acyltransferase, PLTP: Phospholipid Transfer Protein, PON1: Paraoxonase 1.

In addition to the structural distinctions, HDL varies in terms of its diverse properties, encompassing antioxidant, antithrombotic, anti-inflammatory, and anti-apoptotic effects. These properties are potentially linked to their capabilities in preventing LDL oxidation, promoting the regression of atherosclerotic plaques, and preventing atherosclerosis [45]. The main structure of a spherical HDL is illustrated in Figure 1.

## 3. HDL’s Distinct Cargo: Proteins, Hormones, Vitamins, and RNAs

HDL engages with numerous organs, tissues, and cell types, rendering it a complex molecule responsible for transporting bioactive lipids, proteins, hormones, vitamins, and miRNAs to specific target tissues. A particular protein carried by HDL is hemoglobin (Hb), along with its associated binding proteins such as haptoglobin (Hp) and hemopexin (Hx) [46]. Research indicates that these components increase in cases of human coronary heart disease. Another notable protein abundant in HDL is serotransferrin, an iron-binding transport protein. It has been disclosed that hemodialysis can reduce the HDL content of serotransferrin in end-stage renal disease patients [47].

In addition to proteins, HDL is instrumental in transporting hormones, including thyroxine (T4), retinol, estradiol, pregnenolone, and dehydroepiandrosterone. These hormones bind to HDL-apolipoproteins, and their transportation by HDL is crucial for the endocrine and reproductive system, especially during pregnancy. T4, for instance, interacts with several apolipoproteins, such as apo A-II, apo C-I, apo C-II, and apo C-III [48]. HDL also transports esterified hormones like estrogen (estradiol), pregnenolone, and dehydroepiandrosterone.

Vitamins, including carotenoids and vitamin E, represent another category of compounds carried by HDL. HDL has been found to transport a significant percentage of polar carotenoids (lutein: 53% and cryptoxanthin: 39%) and carries a small proportion of nonpolar carotenoids (lycopene: 17%, α-carotene: 26%, and β-carotene: 22%) [49]. Studies in mice suggest that treatment with specific carotenoids increases HDL reverse cholesterol transport capacity and protects against the early development of atherosclerosis [50]. Proteomic studies indicated a relation between HDL and vitamin D-binding protein [51,52], although direct delivery of vitamin D to the liver by HDL has not been conclusively confirmed. HDL is known to transport various forms of retinol, and an association between HDL and plasma retinol-binding protein (PRBP) has been identified [51]. 

The available evidence underscores the presence of diverse RNA entities, notably within the confines of HDL. Among these, one finds tRNA-derived RNA fragments (tRF), RNase P-derived RNA fragments, miRNAs, non-coding, short (~22 nucleotides), and single-stranded RNA molecules pivotal in orchestrating the expression of target genes. These molecules exhibit regulatory capabilities, either by impeding translation or promoting degradation [53,54]. Noteworthy is the discernment that HDL within plasma encapsulates a repertoire of miRNAs. This includes but not limited to miR-33a, miR-30c, miR-92a, miR-122, miR-125a, miR-126, miR-145, miR-146a, miR-150, miR-155, miR-223, miR-378, miR-486, and miR-17/92 [53]. This mRNA suite stands implicated in overseeing indispensable crucial pathways for maintaining cardiovascular homeostasis [55]. Of particular interest, miRNA-33a and miRNA-223 emerge as stalwart defenders against atherosclerosis via modulating HDL cholesterol efflux capacity and lipid metabolism [56,57].

Albeit most of the small molecules found in HDL are likely nonpolar or hydrophobic, there is a large number of polar metabolites on HDL as well, such as pentitol, heneicosanoic acid, and oxalic acid, which are correlated with insulin resistance (HOMA-IR) [58]. Hence, a comprehensive understanding of the extensive functions and roles of HDL in the human body remains an ongoing journey to be fully explored.

## 4. HDL Metabolism and Reverse Cholesterol Transport (RCT)

Apo A-I secretion initiates the intricate process of HDL biogenesis, which is mostly made by the liver (80%) and the intestine (20%). The secreted apo A-I, along with pre-β-1 particles representing small lipid-free apo A-I and lipid-poor yet apo A-I-rich particles, engages with the ATP-binding cassette transporter A1 (ABCA1). This interaction results in cellular phospholipids and cholesterol transfer to apo A-I and pre-β-1 [59], leading to the transformation of lipidated apo A-I into discoidal particles containing unesterified cholesterol, known as nascent HDL (pre-β HDL). Eventually, the discoidal forms evolve into spherical HDL particles by the enzyme lecithin cholesterol acyl transferase (LCAT), which esterifies free cholesterol [59]. Apo A-I has an important role in activating the LCAT enzyme. Apo E and apo A-IV can also contribute to the formation of HDL through an apo A-I-like mechanism.

**Figure 2:**
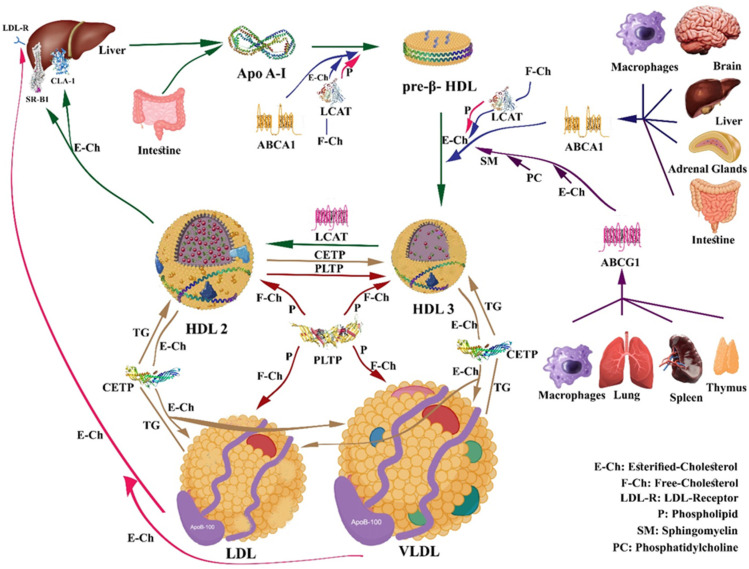
HDL maturation and RCT. ABCA1: ATP-binding cassette sub-family A member 1, ABCG1: ATP-binding cassette sub-family G member 1, Apo A-I: Apolipoprotein A-I, CETP: Cholesterol Ester Transfer Protein, CLA-1: CD36 and LIMPII analogous-1, E-Ch: Esterified Cholesterol, F-Ch: Free Cholesterol, HDL: High-Density Lipoprotein, LCAT: Lecithin–Cholesterol Acyltransferase, LDL: Low-Density Lipoprotein, P: Phospholipid, PLTP: Phospholipid Transfer Protein, SM: Sphingomyelin, SR-B-I: Scavenger Receptor class B type I, TG: Triglycerides, VLDL: Very-Low-Density Lipoprotein.

The reverse cholesterol transport (RCT) mechanism involves several steps mediated by HDL, whereby cholesterol is removed from peripheral tissues and transported to the liver for eventual excretion. In response to the macrophage’s inability to remove excess cholesterol acquired through the enterohepatic pathway or produced endogenously, a return mechanism known as reverse cholesterol transport (RCT) is implemented. This mechanism is initiated with nascent HDL particle formation, which then receives free cholesterol from peripheral tissues, through ATP-binding cassette—A1 (ABCA1) proteins. At this stage, additional receptors, including scavenger receptor class B type I (SR–BI), intervene to facilitate the anchoring of HDL to the plasma membrane as well as membrane cholesterol desorption. Upon maturation, HDL interacts with ATP-binding cassette-G1 proteins (ABCG1) within the intravascular compartment. LCAT, in the presence of its cofactor apo A-I, esterifies free cholesterol. Subsequently, the cholesterol ester transfer protein (CETP) enriches HDL with triglycerides (TG), leading to HDL maturation. The transformation from HDL3 to HDL2 is further facilitated by enzymes like cholesteryl ester transfer protein (CETP) and phospholipid transfer protein (PLTP). Consequently, cholesterol clearance is the main function of HDL. Throughout this process, HDL delivers cholesterol to peripheral tissues and organs, especially the liver via direct and indirect pathways (Figure 2). The direct pathway involves selective cholesterol uptake by hepatocytes through SRB1, while the indirect pathway relies on the effects of CETP to facilitate the exchange of cholesterol esters for TG, between mature HDL and particles containing ApoB, especially VLDL and LDL [60]. The LDL receptor will capture these cholesterol esters in the liver as part of the LDL, eliminating them as cholesterol or bile acids. 

The utilization of plasma TG depends on lipoprotein lipase (LPL) attached to the capillary endothelium, which catalyzes the hydrolytic cleavage of TG into fatty acids. Adipose and skeletal LPL results in triglyceride removal from triglyceride-rich lipoproteins, such as chylomicrons and VLDL. Delipidation of these particles indirectly influences HDL metabolism by altering its constituents. Hepatic lipase also plays a crucial role in HDL metabolism by engaging in the remodeling of both LDL and HDL particles through the hydrolysis of triglycerides and phospholipids. This process results in the formation of smaller and denser particles [61]. A more favorable lipid profile, with lower levels of circulating triglycerides, may support the efficiency of RCT by HDL.

ABCA1 is considered to play a remarkable role in controlling the first phase of RCT, facilitating cholesterol efflux from tissues to nascent HDL [62]. The cardioprotective effects of HDL are greatly related to the ability of apo A-I-containing HDL particles to initiate the removal of excess cholesterol from peripheral tissues. This involves the coaction of HDL with the ABCA1 transporter and LCAT enzyme. Delivering cholesterol to the receptors can occur through both direct and indirect pathways. It has been demonstrated that niacin can selectively inhibit hepatic HDL subfraction-containing apolipoprotein A-I (LP-AI) removal without affecting cholesterol ester uptake. One of the HDL receptors is CLA-1, which stands for CD36, and LIMPII analogous 1. It is a protein expressed in the liver and other tissues, and it belongs to the scavenger receptor class B family, like SR-B1. Northern blot analysis of CLA-1’s tissue distribution in humans has shown that its expression is primarily confined to tissues engaged in highly active cholesterol metabolism, such as the liver and steroidogenic tissues. Considering this discovery, along with the receptor’s ability to bind to both native and modified lipoproteins, it becomes evident that the CLA-1 receptor significantly plays a role in lipid metabolism and may contribute to atherogenesis [63].

Defective lipid efflux in cultivated Tangier fibroblasts is associated with intracellular accumulation of lipids, including cholesterol, ceramide, and cardiolipin, leading to premature senescence.

Besides LDL-lowering therapies, such as PCSK9 (proprotein convertase subtilisin/kexin type 9) inhibitors, two major categories of pharmacological agents have shown promise in improving HDL levels. The first category includes drugs that may increase HDL levels or its components, such as apo A-I. The second category comprises drugs that help to enhance the RCT and macrophage cholesterol efflux. One of the most successful drugs to improve HDL levels, by up to nearly 35%, is Nicotinic acid (niacin, vitamin B3) [64]. Statins, including Rosuvastatin, can increase HDL serum levels by up to 15%, with Rosuvastatin being recognized as the most effective drug in this family [65]. CETP inhibitors are another agent that results in HDL levels elevation and levels of large, less dense HDL2. This action, independent from pre-β-HDL and the ABCA1 pathway, might lead to increased RCT. On the other hand, Fibrates, acting as agonists of peroxisome proliferator-activated receptor (PPAR), can increase HDL levels by 20% and induce RCT as well.

Apabetalone belongs to a distinct class of medications that modestly elevates HDL levels by epigenetically inhibiting the BET (bromodomain and extra terminal) family of proteins. BETs play a significant role in recognizing histone acetylation and exert broad influences on gene expression. Therefore, the mechanism involves epigenetic modification, altering apo A-I transcription. The resulting effect on increasing HDL levels is about 5 to 10 percent. Ongoing studies are exploring HDL infusion therapy, involving the direct infusion of exogenous HDL, and Recombinant LCAT, a medication designed to enhance the esterification of cholesterol in HDL [66].

The breakdown of apo A-I by proteases is a pivotal facet in HDL catabolism, as it leads to the release of free cholesterol from the HDL. This cholesterol becomes amenable to cellular uptake or elimination from the body, thereby safeguarding cholesterol homeostasis. However, excessive protease activity can lead to accelerated degradation of HDL, affecting cholesterol homeostasis and thereby contributing to the development of CVD. Mast cell tryptases and chymases actively degrade HDL, yielding functionally impaired particles that are unable to initiate cholesterol efflux from the arterial wall [67].

The mechanism orchestrating HDL catabolism encompasses several processes: (1) Hepatic uptake: HDL can be taken up by the liver through the SR-B receptor. Once internalized within the liver, the HDL is catabolized, and its components can be either metabolized or expelled from the body. HDL can be incorporated into the bile and ultimately eliminated from the body through feces. (2) Enzymatic degradation: HDL can be broken down by enzymes such as lipases and proteases, which hydrolyze the lipids and proteins constituting HDL. This process transpires in various tissues throughout the body. (3) Renal clearance: HDL can also be cleared from the body through the kidneys. 

Mature spherical α-HDL are removed from the plasma via complete catabolism and cholesterol uptake by the liver, kidney, adrenal gland, and ovaries selectively. The SR-BI receptor, representing the principal catabolic system, mediates the process of selective cholesterol uptake [68]. During this SR-BI-mediated process, lipids are transported from the HDL core to tissues without degradation of the particle. Small, dense HDL particles, released through interaction with SR-BI are remodeled in the plasma, forming HDL2 particles that re-enter the previously elucidated metabolism pathway. In ABCA1 knockout mice, specifically in the liver or whole body, the catabolism of plasma HDL, plus the fractional catabolic rate of HDL was increased, predominantly by the liver and to a lesser extent by the kidney and the adrenal [69]. Also, in ABCA1-deficient mice, lipidated apo A-I particles or even preβ-HDL were unable to mature and were rapidly catabolized by the kidney. It is suggested that HL decreases the size of α-migrating HDL and enhances the rate of apo A-I catabolism [70], in addition to its action of promoting selective HDL3 cholesterol ester uptake independent from SR-BI [71].

Endothelial lipase (EL) is another enzyme that has been illustrated to play a significant role in HDL catabolism. For example, the expression of EL in mice leads to a dose-dependent increase in post-heparin plasma phospholipase activity, the catabolism rate of HDL-apolipoprotein, and the uptake of apo A-I in both the kidney and liver [72]. 

It has been demonstrated that the mean plasma residence time of radiolabeled HDL_2_ and HDL_3_ is 6 days in normal subjects. Complete catabolism of HDL (lipid-free and poorly lapidated Apo A-I) has been illustrated, mostly in the kidney. This process is initiated by endocytosis, followed by lysosomal degradation of HDL or apo A-Ⅰ. The kidney cortex is a major organ of catabolism for lipid-free and poorly lipidated apo A-I through a concerted action of glomerular filtration, tubular reabsorption, and intracellular degradation of free apo A-I [73]. The role of the kidney in HDL catabolism is related to Cubilin, which has an important role in binding to apo A-I or HDL. Undoubtedly, HDL particles are too large to cross the glomerular filtration barrier, and hence the megalin and cubilin/amnionless protein receptor system is only exposed to filtering lipid-free or poorly lipidated apo A-I. As a result, megalin and cubilin/amnionless can affect the overall HDL metabolism [74,75]. Although the kidney is not regarded as a central organ in the catabolism of lipoproteins, it plays an important role in the degradation of lipid-poor apo A-I through the cubilin function [76]. 

Meanwhile, it seems that renal catabolism has little impact on total HDL levels in plasma. According to several studies, discoidal and small-size HDL particles and LCAT associated with them can be rapidly catabolized via the kidney. This process can result in LCAT insufficiency and a subsequent decrease in plasma HDL levels [77,78]. However, the proposed importance of LCAT in the process of RCT has not been firmly confirmed. The absence of functional LCAT activity does not necessarily lead to a great defect in RCT [79], probably because much RCT may occur as unesterified cholesterol. Certainly, in humans, unesterified cholesterol in HDL can be instantly transferred to the liver and secreted in bile [80]. Nevertheless, further studies are needed to determine how the percentage of the HDL lipid components such as esterified or unesterified cholesterol may modify the HDL structure, inducing an increase or decrease in their capacity to mediate RCT and to reduce the process of atherosclerosis.

Overall, all the mentioned mechanisms of HDL catabolism play an important role in maintaining cholesterol homeostasis in the body and preventing the accumulation of excess cholesterol in peripheral tissues.

## 5. HDL Interaction with Major Proteins and Receptors Involved in Cholesterol Efflux

### 5.1. HDL and ABCA1 Transporter

ABCA1, a member of the superfamily of ATP-binding cassette (ABC) transporters, is a ubiquitous membrane-associated protein that is also expressed in every human cell, and in large quantities in the liver, macrophages, brain, and various other tissues. In hepatocytes, ABCA1 is found only on the basolateral surface [81]. ABCA1 is also considered a cholesterol efflux regulatory protein since it facilitates the efflux of free or unesterified cholesterol to lipid-free or lipid-poor apo A-I in the plasma, but not to spherical HDL particles [82]. Inactivating mutations or the absence of apo A-I, ABCA1, and LCAT prevent the formation of HDL containing apo A-I [83]. Another considerable role of ABCA1, which was shown in bone marrow transplantation experiments, is controlling macrophage recruitment to the tissues [84].

Lipid-free apo A-I and discoidal HDL particles preferentially receive cholesterol efflux through ABCA1, whereas spherical, lipid-enriched HDL particles accept cholesterol through ATP-binding cassette sub-family G member 1 (ABCG1) and scavenger receptor-BI (SR-BI) [85]. 

Moreover, studies have demonstrated that ABCA1 can improve the fluidity of the plasma membrane through its intrinsic molecular function, leading to an increased release of microparticles in the absence of exogenous acceptors, resulting in moderate levels of lipid efflux [86].

ABCA1 features two substantial extracellular loops that are critical for binding with apolipoprotein A-I (apo A-I). While ABCA1 is a full-size ABC transporter, ABCG1 is a half-type ABC transporter, comprising a single nucleotide-binding domain and a single transmembrane domain. ABCG1 functions by forming a homodimer configuration.

### 5.2. HDL and ABCG1 Transporter

ABCG1, a member of the ABC family of half transporters, is expressed in the spleen, thymus, lung, and brain. It has been illustrated that it is localized in the plasma membrane, Golgi, and recycling endosomes. ABCA1 and ABCG1 are responsible for transporting cholesterol and preventing its buildup in macrophages. ABCA1 facilitates the movement of cholesterol and phosphatidylcholine to lipid-free apo A-I, resulting in the creation of nascent HDL (preβ-HDL). In contrast, ABCG1 supports the transfer of cholesterol, phosphatidylcholine, and sphingomyelin to both nascent HDL and mature HDL particles. ABCG1 has been shown to mediate cholesterol efflux to mature HDL particles from other types of cells as well, such as adipocytes [87] and human placental endothelial cells, and it might facilitate the transportation of maternal cholesterol to the fetus [88]. Excessive expression of ABCG1 can enhance the cholesterol efflux from various cells to HDL but not to lipid-free apo A-I [89,90,91]. Based on some studies, it has been shown that HDL obtained from individuals who were CETP-deficient or patients who were treated with the CETP inhibitors (torcetrapib or anacetrapib) had an elevated ability to promote cholesterol efflux via ABCG1 [92]. ABCG1 can protect cells from apoptosis by promoting efflux of 7-ketocholesterol and related oxysterols from macrophages and endothelial cells to HDL [93]. 

Studies related to genetics in humans discovered various functions of ABCG1 associated with increased risk of CAD, revealing a notable role of ABCG1 in protecting from atherosclerosis and CVD [94]. The absence of ABCG1 might reduce the efficiency of HDL-mediated cholesterol removal, potentially causing an increase in inflammatory signal transmission. This scenario could contribute to the excessive buildup of lipids. Also, the lack of ABCG1 could influence raft domains, specialized regions abundant in cholesterol and sphingomyelin that play a vital role in signal transduction, due to its role in transporting these lipids. Genetic polymorphisms within ABCA1 or ABCG1 have been associated with susceptibility to atherosclerosis [9]. Research suggests that diminished functions of ABCA1 and ABCG1 result in cholesterol accumulation within cells and contribute to atherosclerosis development. Conversely, elevated activities of ABCA1 and ABCG1 are anticipated to alleviate cholesterol burdens. Indeed, the overexpression of either ABCA1 or ABCG1 heightens HDL levels and confers protection against atherosclerosis [95].

### 5.3. HDL and SR-BI Receptor

SR-BI plays a crucial role in the nuanced orchestration of plasma HDL, intricately influencing its composition and concentration [96]. Beyond its regulatory prowess, SR-BI stands as a stalwart guardian, affording protection against atherosclerosis in various murine models. Nevertheless, the role of SR-BI in the liver’s selective uptake process of HDL cholesteryl ester (CE) is intricate and necessities the functions of PDZK1, a liver-specific protein, endowed with four PDZ domains attuned to the C-terminal region of SR-BI. The interaction of PDZK1 with the C-terminal region of SR-BI, post-transcriptionally, adjusts the localization and stability of SR-BI [97]. Hepatic PDZK1 inactivation can significantly affect plasma HDL metabolism and structure, leading to occlusive atherosclerosis in mice deficient in both apo E and PDZK1 [98]. The envisaged mechanism underscoring SR-BI’s facilitation of selective CE uptake unfolds with HDL binding to the hepatic SR-BI. Herein, SR-BI generates a channel, ushering CE to pass the cell membrane based on the concentration gradient, enabling its ingress into hepatocytes. During this process, HDL gives its CE to hepatocytes without the simultaneous uptake and degradation of the whole HDL particle [76], which allows the generation of new preβ-HDL particles that can be used in the next RCT pathway.

HDL with SR-BI interactions in endothelial cells initiates signaling mechanisms that involve activation of endothelial nitric oxide synthase (eNOS) and eventually nitric oxide release which causes vasodilation [99]. Based on a human study, patients with a P297S substitution in SR-BI (which is a functional mutation) had increased HDL levels and reduced adrenal steroidogenesis and dysfunctional platelets, yet paradoxically evaded atherosclerosis development [100]. Moreover, HDL derived from these subjects manifests a reduced ability to amplify cholesterol efflux from macrophages.

In murine physiology, SR-IB emerges as a substantial factor in the synthesis of steroid hormones within the steroidogenic tissues, in addition to its role in the control of HDL plasma levels [101,102,103]. SR-BI deficiency in mice results in reduced HDL clearance, concurrent elevation in plasma cholesterol, and the presence of large-size abnormal apo E-enriched particles in HDL, IDL, and LDL [96]. This deficiency can decrease the CE stores of steroidogenic tissues, and it reduces biliary cholesterol secretion by nearly 50%. Regarding bile acids, SR-BI deficiency did not impact the secretion of the pool size or fecal secretion, nor did it affect cholesterol absorption in the intestine [96,104]. Moreover, the lack of functional SR-BI has been discovered to lead to defective maturation of oocytes and erythrocytes, attributable to cholesterol accumulation in the plasma membrane of progenitor cells [105].

### 5.4. HDL and ectoF1-ATPase Receptor

In the culmination of the RCT process, hepatocytes undertake the vital step of endocytosing HDL. Once internalized, cholesterol derived from HDL can be converted to bile acids, secreted into the bile, or secreted via new lipoproteins synthesized in the liver. HDL is the primary source of biliary sterols and phospholipids in humans [106]. Notably, recent revelations emphasize the pivotal role of F1-ATPase as an apo A-I receptor [107]. F1-ATPase is the catalytic part of ATP synthase, an enzymatic complex classically located within mitochondria and coupled to the mitochondrial respiratory chain. Nevertheless, several subunits of this complex were recently found ectopically expressed on the plasma membrane of various cells, such as hepatocytes and endothelial cells [107].

Binding the ectopic cell surface F1-ATPase (henceforth ecto-F1-ATPase) to apo A-I stimulates the hydrolysis of extracellular ATP into ADP and phosphate, a process that is prevented by the F1-ATPase inhibitor, Inhibitory Factor 1 (IF1). The released ADP, contingent on the cell type, mediates the activation of distinct ADP-responsive P2Y receptors (P2Y ADP receptors), which translates into distinct cellular processes. For instance, ecto-F1-ATPase activity in hepatocytes is specifically coupled to the P2Y13-ADP receptor, facilitating HDL holoparticle endocytosis [107]. 

On endothelial cells, ecto-F1-ATPase activation by HDL-apo A-I inhibits apoptosis and promotes proliferation [107]. The endothelial cell ecto-F1-ATPase has been characterized as being associated with the P2Y12-ADP receptor and contributing to HDL uptake and the uptake of lipid-free apo A-I. Moreover, apo-A1 enhances endothelial nitric oxide (NO) production, thereby controlling vascular tone through a process that requires activation of the ecto-F1-ATPase/P2Y1 pathway by apo-A1 [11]. The inhibitor factor 1 (IF1) was recently identified in human serum and found to be positively and independently associated with HDL and apo A-I levels while exhibiting a negative correlation with the severity of coronary heart diseases (CHD) [107].

### 5.5. HDL and LCAT

The maturation and transition of pre-β HDL into α-HDL hinges on the conversion of incorporated free cholesterol into cholesterol esters, a more hydrophobic form [108]. As nascent HDL circulates through the bloodstream, apo A-I activates the plasma LCAT. Synthesized by the liver, and to a much lesser proportion by the brain and the testis [109], LCAT is an enzyme that activates apo A-I, catalyzes the transfer of the sn-2 acyl group from lecithin to free cholesterol in nascent HDL (discoidal and spherical HDL), and forms cholesterol esters and lysolecithin. Lysolecithin is subsequently removed via circulating albumin, facilitating the transfer of the newly formed cholesterol esters into the hydrophobic core of the discoid structure of nascent HDL. This dynamic process leads to the expulsion of the double phospholipid layer, ultimately transforming the molecular shape into a spherical α-HDL [108]. LCAT also catalyzes the reverse reaction of lysolecithin to lecithin esterification [110]. In humans, genetic LCAT deficiency has been associated with the accumulation of unesterified cholesterol in some organs. Additionally, LCAT deficiency notably decreased HDL levels and fast catabolism of cholesterol esters-poor apo A-I [111]. The disease caused by LCAT deficiency is distinguished by two discrete phenotypes: (1) familial LCAT deficiency (FLD) and (2) Fisheye disease (FED). Regarding FLD, the alpha and beta LCAT activity is lost, causing intensely low plasma HDL, hemolytic anemia, proteinuria, renal failure, and premature corneal opacification [112]. Conversely, FED involves the loss of only alpha LCAT activity, allowing cholesterol esterification in LDL and VLDL but not in HDL. Consequently, the HDL particles in FED carry only 20 % cholesteryl ester, dissimilarly to 75% to 80 % in HDL of the control group. Patients with FED have undergone corneal opacities and reduced HDL levels, but they are clear of the renal implications observed in FLD [113]. Moreover, plasma analysis of patients with complete LCAT deficiency, using two-dimensional electrophoresis, showed a high proportion of the presence of a small α-HDL subgroup [83]. 

Mouse knockdown experiments targeting LCAT have demonstrated that diminished LCAT activity has a more pronounced impact on atherogenic apo B-containing lipoproteins compared to its effect on HDL reduction in the progression of atherosclerotic lesions [12]. In experiments involving rabbits, LCAT overexpression led to elevated plasma levels of HDL and decreased lipoprotein B-containing lipoproteins. These changes were correlated with a substantial reduction in atherosclerosis. The findings of studies examining the link between LCAT deficiency and atherosclerosis in humans have yielded varying and inconclusive results [12,114]. Overall, the role of increased or decreased LCAT activity in coronary heart disease remains uncertain, likely owing to the intricate nature of lipoprotein and lipid biosynthesis and breakdown processes. Additional research is required to elucidate the mechanism by which LCAT interacts with lipoproteins and its involvement in the metabolic process of RCT. Therefore, this research could help enhance the investigation of LCAT as a potential therapeutic agent influencing HDL and RCT.

### 5.6. HDL and CETP

The CETP enzyme catalyzes the transportation of cholesterol esters from α-HDL to VLDL and chylomicrons, circulating in the bloodstream, in exchange for phospholipids. Therefore, the phospholipid/cholesterol esters content ratio in α-HDL increases. Patients with CETP deficiency show increased levels of HDL [115]. Additionally, great levels of plasma CETP activity can reduce HDL in humans [116]. In response to these findings, efforts have been directed towards the development of CETP inhibitor drugs. During the first phase III RCT of a CETP inhibitor (ILLUMINATE), it was shown that torcetrapib raised levels of plasma HDL by about 72% and decreased plasma LDL levels by 25% compared with a placebo in patients at high risk of CVD (*n* = 15,067) [117]. However, the trial was terminated early due to a 25% higher risk of major vascular events in the torcetrapib group, linked to elevated systolic blood pressure. In the dal-OUTCOMES trial, which included 15,871 patients with a recent acute coronary syndrome, the CETP inhibitor dalcetrapib elevated HDL levels by 31–40% from the baseline but had a slight effect on LDL levels. This trial was also terminated early due to futility, with a hazard ratio (HR) for the primary endpoint of major vascular events of 1.04 [118]. Similar results have been shown in ACCELERATE and REVEAL trials [119]. A new CETP inhibitor, obicetrapib, which has demonstrated excellent safety and tolerability in phase 1 and 2 trials (ROSE2 or Systematic Coronary Risk Evaluation ROSE2 study) [120], is being studied in a phase 3 trial and is anticipated to be a promising agent in the prevention of CVDs. 

Mipomersen is an antisense oligonucleotide inhibitor designed to target apolipoprotein B-100 (apoB-100) mRNA, administered via subcutaneous injection. Following administration, mipomersen induces the selective degradation of apoB-100 mRNA, leading to the inhibition of protein translation. This process ultimately yields significant reductions in LDL-C and other lipoprotein levels, with no observed change in HDL-C levels. Mipomersen has received approval for the treatment of homozygous familial hypercholesterolemia (FH) [121].

HDL remodeling by the hepatic lipase (HL) action and endothelial lipase (EL) involves hydrolysis of residual triglycerides and some phospholipids of HDL. This process leads to the conversion of HDL2 to HDL3 and preβ-HDL [122], which increases HDL catabolism. Preβ-HDL formation also requires the functions of apolipoprotein M (apo M) [123]. Analysis of HL in transgenic rabbits showed that HL reduces the size of α-migrating HDL and increases the rate of catabolism of apo A-I. A portion of the cholesteryl esters that were formed by LCAT can be transferred to VLDL, LDL, and IDL by CETP [124]. 

Prospective studies have revealed a decline in HDL levels with age in both men and women. Genetic factors that may be responsible for higher HDL levels and larger HDL particle sizes in older people include genetic heterogeneity in CETP and apo C-Ⅲ. Moreover, studies have illustrated that CETP genotypes associated with moderate inhibition of CETP activity and slightly higher HDL levels show a converse association with CVD risk and preservation of cognitive functions. Mildly decreased levels of apo C-Ⅲ, which is an endogenous inhibitor of LPL, can lead to increased triglyceride clearance and a lower incidence of subclinical atherosclerosis [125]. Therefore, inhibition of apo C-III might be a promising strategy in managing severe hypertriglyceridemia and, more generally, a new approach to CHD prevention in patients with high plasma triglycerides [125]. Conversely, excessive CETP inhibition might reduce RCT, and certain CETP mutants may even induce a proatherogenic state [126]. Eventually, the overall role of CETP mutants in longevity remains unclear, and complete apo C-Ⅲ inhibition poses challenges due to the enhanced formation and uptake of free fatty acids resulting in obesity and insulin resistance [126].

### 5.7. HDL and PLTP

PLTP belongs to the lipid transfer proteins family, alongside CE transfer protein (CETP), lipopolysaccharide-binding protein (LBP), and bactericidal/permeability-increasing protein (BPI) [127]. PLTP exhibits a broad spectrum of functions, facilitating the transfer of free cholesterol, phospholipids, diacylglycerol, cerebroside, α-tocopherol, and lipopolysaccharides among lipoproteins and between lipoproteins and cells. PLTP is expressed ubiquitously, with varying degrees across different organs. The liver and adipose tissue play a particularly crucial role, with higher expression levels, especially in larger amounts. Notably, PLTP is expressed in macrophages and atherosclerotic lesions in high amounts. Adipose tissue expresses higher PLTP than the liver [128]. As mentioned previously, PLTP can not only transfer phospholipids but also unesterified cholesterol [129], which is more abundant than cholesteryl esters in adipose tissue. In other words, PLTP can transfer the phospholipids from VLDL or IDL to the HDL particles during lipolysis to generate HDL2 and can also convert HDL3 to HDL2 and preβ-HDL [130].

Studies have associated active plasma PLTP with apo A-I, while slow activity is linked to apo E [131]. Nevertheless, the role of these two forms of PLTP in the circulation is still unclear. Moreover, it is unknown whether animals like mice and rabbits possess two forms of PLTP. These findings suggest that PLTP might have lipid transfer–independent activity [132]. For example, PLTP deficiency significantly decreases brain vitamin E content and is associated with increased anxiety in mice [133]. 

In the context of its relationship with HDL, plasma PLTP mediates the phospholipids transition from apo B-containing triglyceride-rich lipoproteins into HDL and engages in the overall exchange of phospholipids between lipoproteins. Functioning as a putative fusion factor, PLTP assists in enlarging HDL particles [134] with a core rich in TG, thereby enhancing the fusion process [135]. Overexpressing human PLTP in mice showed an increase in PLTP activity in plasma. Therefore, this resulted in a 30–40% decrease in plasma HDL cholesterol levels, but a 2- to 3-fold increase in the preβ-HDL formation [136]. In a study by Yazdanyar et al., it was found that a deficiency of liver-specific PLTP significantly reduces HDL and apo A-I levels [137]. In conclusion, PLTP overexpression leads to a significant reduction in HDL levels but increases preβ-HDL concentration [132]. On the other hand, studies indicated that PLTP plays an essential role in specifying the plasma HDL particles’ size, lipid composition, and inflammatory index [138]. 

### 5.8. HDL and Paraoxonase 1 (PON1)

Paraoxonase 1 (PON1) is an enzyme that is closely associated with HDL and is believed to contribute to the antioxidant and anti-inflammatory properties of HDL. Low levels of both HDL and PON1 activity have been associated with an increased risk of CVD, whereas high levels of both HDL and PON1 activity have been demonstrated to be protective [139]. However, the exact relationship between HDL, PON1, and CVD risk is complex and not fully understood. Several studies suggest that PON1 activity may serve as a more accurate predictor of CVD risk than HDL levels alone. PON1 activity was demonstrated as a significant predictor of future cardiovascular events, independent of HDL levels [140]. Earlier findings from our team indicated that the overexpression of human PON1 stimulates RCT and enhances HDL efflux capacity [141].

PON1 is a member of a family of proteins which includes PON2 and PON3 as well. PON1 is primarily synthesized in the liver and a portion is subsequently secreted into the plasma, where it associates with HDL. The most important function of PON1 appears to be the metabolism of bioactive lipids contained within oxidized LDL as well as HDL [142]. Also, PON1 protects HDL phospholipids from oxidation [143], and it can metabolize several drugs and pro-drugs through its lactonase activity [142,144]. 

PON2 and PON3 both have antioxidant properties. While PON3, similarly to PON1, is primarily expressed in the liver and is associated with HDL, PON2 is more widely distributed [145].

Various exogenous compounds can modulate PON1, including environmental chemicals, drugs, smoking, alcohol, and diet, such as a high-fat diet or compounds like olive oil. The results from these studies suggest that the fatty acid composition of phospholipids might affect PON1 activity. For example, these animal findings were in part confirmed in a human study where 14 individuals with type 2 diabetes received meals rich in thermally stressed olive oil or safflower oil. Only the olive oil meal elevated serum PON1 activity, and it is notable that the effect was most pronounced in women [146]. Omega-3 polyunsaturated fatty acid administration for eight weeks caused a significant 10% increase in PON1 plasma concentration in hyperlipidemia patients [147]. 

Studies have illustrated that older people exhibit a significant decrease in serum PON1 levels compared to adults. This might be associated with oxidative stress as explained earlier. A study published by Bhattacharyya et al. [148] indicated that PON1 has an important role in age and oxidative stress-related diseases such as diabetes and heart diseases [148]. Moreover, in recent years, it has become increasingly apparent that during disease development, HDL becomes dysfunctional [149]. Therefore, much larger studies and mechanistic investigations of the role of PON1 are needed to elucidate the exact relationship between PON1 polymorphisms and CVDs, specifically in humans.

Eventually, paying more attention to PON1 activity as a predictor and indicator of chronic diseases, such as CVDs, and factors that may improve its function through appropriate interventions, seems necessary, especially in elderly people. 

## 6. Principal HDL Functions Related to CVDs

### 6.1. Antioxidant/Pro-Oxidant Properties of HDL

HDL plays a crucial role in protecting against oxidative damage, particularly preventing LDL from ROS-induced oxidative damage. The antioxidant activity of HDL is in part attributed to PON1 action [150]. PON1 may exert its antioxidant effect via hydrolyzing and detoxifying oxidized lipids found in oxLDL, a major contributor to atherosclerosis development. By breaking down these oxidized lipids, PON1 helps prevent the formation of foam cells, thereby mitigating the risk of atherosclerosis. Numerous studies highlight the protective effects of HDL and PON1 against atherosclerosis and CVDs. Individuals with low levels of HDL or PON1 exhibit a higher risk of developing CVD, while interventions that increase HDL levels or PON1 activity have been associated with a reduced risk [151].

Lipid hydroperoxides are reactive compounds that are formed when polyunsaturated fatty acids (PUFAs) are oxidized. Lipid hydroperoxides formed within LDL migrate to its outer layer as a result of their higher hydrophilicity [152]. This makes it easier for HDL to remove these oxidized lipids more effectively through lipid exchange [153]. During this process, HDL removes lipid hydroperoxides from the surface of LDL, while at the same time transferring cholesterol esters to LDL. This process is facilitated by enzymes contained within HDL such as LCAT and PLTP. Within HDL, these oxidized lipids are inactivated through the action of PON1, among other enzymes, or transferred to the liver [154]. There are other shreds of evidence suggesting that HDL metabolizes these highly active lipid hydroperoxides, preventing their accumulation, and afterwards inhibiting the formation of the atherogenic structure of LDL [155].

Apo A-I, another key HDL protein, contributes to HDL’s antioxidant activity and might have a main role in HDL-mediated antioxidative functions. Apo A-I can reduce lipid hydroperoxides (LOOH) into redox-inactive lipid hydroxides (LOH), thereby impeding chain reactions of lipid peroxidation. Other HDL apolipoproteins, including apo E, apo J, apo A-II, and apo A-IV [156] are believed to have a role in HDL-mediated protection against LDL peroxidation [157]. Apo E has been shown to interact with LDL, inhibiting its oxidation directly, as well as enhancing the antioxidant properties of HDL by promoting the activity of PON1. Apo J, also known as clusterin, exhibits anti-oxidative effects against LDL oxidation by binding to oxidized LDL particles, reducing their ability to interact with macrophages and form foam cells. Additionally, apo J helps neutralize free radicals that can contribute to LDL oxidation [158]. 

HDL can potentially integrate lipid peroxidation products and phospholipids containing hydroperoxides, which are produced by oxidized LDL. Moreover, HDL plays a role in hydrolyzing oxidized phospholipids, such as F2-isoprostanes, which are formed during the oxidative modification of LDL. On the contrary, oxidized HDL might trigger the generation of reactive oxygen species (ROS), increasing the risk of CVDs by elevating the expression of pro-inflammatory genes, such as tumor necrosis factor-alpha (TNF-α), cyclooxygenase 2, and plasminogen activator inhibitor-1 (PAI-1) [14]. Adding to this, the enrichment of HDL with triglycerides, MPO, phospholipase A2, ceruloplasmin, and serum amyloid A (SAA) diminishes its antioxidant capabilities and fosters the formation of pro-oxidant HDL. Studies have shown that oxidized HDL loses its ability to facilitate cholesterol efflux from foam cells [13]. 

### 6.2. Anti-Inflammatory/Pro-Inflammatory and Immunomodulatory Effects of HDL

Although HDL has always been reported as a protective and anti-inflammatory particle, there is also evidence attributed to some pro-inflammatory effects of HDL [159]. Assessed by its capability to inhibit TNF-α-induced VCAM-1 expression, the anti-inflammatory potential of HDL has been autonomously linked to cardiovascular incidence within the broader population [160].

The anti-inflammatory effects of HDL are primarily attributed to its ability to modulate immune cell function and inhibit the production of pro-inflammatory cytokines [161]. It is reported that HDL decreases pro-inflammatory cytokines by affecting their stream cascades, increasing the induction of ATF3 (activating transcription factor 3) and suppressing TLR/NFκB (nuclear factor-κ light-chain enhancer of activated B cells) signaling [162]. 

It is also reported that HDL affects the interferon-gamma (IFNγ) signaling pathways. Studies have shown that HDL can have both positive and negative effects on IFNγ, depending on the context [163]. On the one hand, HDL can enhance the production and activity of IFNγ, which can help to promote an effective immune response. On the other hand, HDL can also suppress the production of IFNγ, which could have negative consequences for the immune response [164]. HDL has been also shown to suppress the activation and migration of monocytes and macrophages, which are immune cells that play a key role in the development and persistency of inflammation [165,166].

Reconstituted HDL also inhibits the expression of adhesion molecules, such as CD11b, on endothelial cells, which are responsible for attracting immune cells to sites of inflammation [167]. This prevents the migration of immune cells to inflamed tissues and reduces the overall inflammatory response. Moreover, HDL can directly bind to and neutralize pro-inflammatory molecules such as lipopolysaccharides (LPS), which are components of bacterial cell walls that can induce inflammation [168]. Finally, HDL can promote the production of anti-inflammatory molecules such as interleukin-10 (IL-10) and transforming growth factor-beta (TGF-β), which help to dampen the immune response [169].

In addition to apo J, the anti-inflammatory action of HDL is also linked to its sphingosine-1-phosphate (S1P) content. In the most recent developments, sphingosine-1-phosphate receptors (S1PR) have gained significant prominence as potential target receptors for HDL. This interest stems from the fact that a substantial portion, approximately 50% to 70%, of circulating S1P is transported by HDL. Activation of S1PR1 and S1PR3 through HDL exerts protective effects on endothelial cells, diminishing inflammation and apoptosis. More precisely, the enrichment of S1P in HDL effectively hinders apoptosis induced by oxidized low-density lipoprotein while also enhancing the production of nitric oxide (NO) [170].

Conversely, in a state of systemic inflammation, HDLs are transformed into dysfunctional lipoproteins that can induce an increase in oxidized lipids and proteins, further amplifying their pro-inflammatory condition. These dysfunctional HDLs release pro-inflammatory cytokines that stimulate the recruitment of monocytes into arterial walls. This process ultimately leads to the accumulation of macrophages. Dysfunctional HDLs can also be generated by myeloperoxidase (MPO). When released from macrophages within atherosclerotic lesions, MPO induces oxidative harm to apo A-I. Notably, HDLs obtained from atherosclerotic lesions harbor a substantial amount of MPO-modified proteins, including nitrated and chlorinated apo A-I [66,171].

At present, conventional lipid measurements conducted in laboratories have evolved into a potentially misleading scale of an individual’s health status. Given the current understanding, it becomes evident that, when evaluating the role of HDL within the body, qualitative assessment holds greater significance than quantitative one. This is due to the inherent structural and compositional diversity within HDL, which in turn contributes to its wide-ranging functional capabilities. As a result, HDL’s actions can vary substantially based on different scenarios. Consequently, delving deeper into dysfunctional HDL, which emerges as both a causal factor and a manifestation of numerous ailments, particularly atherosclerosis, stands to offer valuable insights through further research.

## 7. Aging, HDL, and CVD

Foam cells are specialized cells that form when macrophages in the circulation ingest large amounts of small LDL particles. When LDLs accumulate in the arterial wall and undergo oxidative modifications, these oxidized LDL are taken up by macrophages through a process called monocyte-macrophage LDL receptors [172], resulting in cholesterol and lipids accumulation within macrophages, leading to the formation of foam cells [173]. The accumulation of foam cells is a hallmark of early atherosclerotic lesions, a precursor to CVDs. Foam cells contribute to the progression of atherosclerosis by secreting pro-inflammatory cytokines, promoting oxidative stress. 

The reduction in the clearance of chylomicrons and their remnants is associated with atherosclerosis and coronary artery disease (CAD), and it has been demonstrated that the postprandial removal of remnants is slower in elderly individuals compared to youths [174]. In addition, in the elderly, postprandial plasma TG values exhibited elevated peaks of triglycerides, along with a more prolonged return to the triglyceride levels present before the consumption of the fat test meal. In contrast, in a recent study comparing elderly individuals (aged 66 ± 4 years) with young individuals (aged 24 ± 3 years), the chylomicron-like emulsion kinetics approach was employed. The study revealed that the elderly participants exhibited a slower removal rate of chylomicron remnants. However, there was no discernible difference in the removal of emulsion triglycerides, which represents the lipolysis process [174].

Aging can increase the incidence of CVDs [175], while atherosclerosis is the underlying histo-pathological process. One of the most important initiating factors is hypercholesterolemia, which is associated with an increased susceptibility of LDL to oxidation [176]. Indeed, oxidized LDL triggers an inflammatory process, which conclusively leads to the formation of atherosclerotic plaques [177]. Furthermore, it has been shown that LDL and HDL have an increased susceptibility to oxidation with age [178,179]. In addition to oxidation, glycation is another age-related change in the composition of lipoproteins. Glycation has the potential to obstruct the recognition and clearance of lipoproteins by the cellular receptors that are tasked with their uptake and removal from circulation. Another probable mechanism for the reduction in the plasma clearance of the lipoproteins and the increase in LDL-cholesterol levels (hypercholesterolemia) is diminished expression of LDL receptors in the elderly population [180]. Moreover, aging affects HDL composition and function, and many pathological age-related changes in lipid profiles were reported in various scientific investigations.

The antiatherogenic property is one of the best-known features of HDL, and has been demonstrated to be related to PON1. Studies have revealed that PON1 activity can be affected by aging, probably via the development of an inflammatory status leading to an increase in circulating acute phase reactant, C-reactive protein (CRP). Ultimately, it can be inferred that the decrease in PON1 activity might be the result of the development of oxidative stress conditions with aging and also the increased HDL susceptibility to oxidation among the elderly population [175,181]. It has been revealed that functional HDL can undergo impairments by various stressors, like aging, infection, bad eating habits, smoking, and pollutants. At worst, HDL has demonstrated a transposition of apo A-I, a reduction in cholesterol, and augmentation with SAA, MPO, TG, and apo C-Ⅲ. 

The effects of aging on HDL levels and function are not completely understood, but some research suggests that as people age, their HDL levels may decrease or become less effective at removing cholesterol from the blood vessels [126,182]. However, other studies showed that HDL levels in plasma increase with aging [183] or decrease by a small amount [184]. However, it is still largely known that aging alters HDL composition, resulting in functional impairments that might lead to the onset/progression of CVDs [185]. Based on our previous study, HDL from the elderly presents a reduced capacity to promote cholesterol efflux, mainly via the ABCA1 pathway, and this may explain the increase in the incidence of CVDs observed during aging [27]. Moreover, Annelies and colleagues reported that the increased mortality by stroke and coronary artery disease in old age is due to low HDL levels. Also, oxidative modifications of apo A-I that might occur with aging may affect the ABCA1-apoA-I interaction, leading to the reduction in cholesterol efflux [27]. SDS-PAGE analysis demonstrated a decrease in the apo A-I band intensity in the HDL of elderly individuals compared with the HDL of young individuals without significant changes in apo A1 plasma concentration [175]. This suggests a structural alteration of apo A1 which may affect cholesterol efflux capacity [175]. Therefore, there is a crucial need for more studies to clarify the specific differences between HDL in the geriatric population and young people.

HDL isolated from elderly individuals illustrated decreased free cholesterol content and apo E, alongside being enriched in sphingomyelin. Apo E is produced by cholesterol-loaded macrophages, where it can improve cholesterol efflux throughout its secretion. Apo E is part of a gene cluster that is induced in macrophages by cholesterol-sensing nuclear receptors that protect against atherosclerosis in mice [186]. Interestingly, recent studies reported that chronic inflammation can notably remodel HDL composition associated with increased serum amyloid A (SAA) and apo C-Ⅲ content in HDL [187,188]. It can be inferred that alterations in the composition of HDL induced by aging may play essential roles in inflammatory responses and lipid metabolism.

Studies focused on the RCT function of HDL observed that the lipid content of HDL from aged persons is more promptly taken up by macrophages [185]. Thought-provokingly, it has been displayed that serum PLTP activity was increased in older communities and correlated with macrophage uptake of HDL-lipids. These results are consistent with previous findings illustrating that increased serum PLTP activity can change the structure and composition of HDL which leads to increased cellular uptake of HDL lipids [189,190]. High expression of PLTP has been indicated to be associated with various pathologies such as insulin resistance, obesity, and type 2 diabetes. It is suggested that the increased uptake of lipids from HDL by macrophages may also contribute to CVD risk in these people [185]. 

The aging process can decrease LCAT activity and cholesterol efflux. Also, it has been illustrated that aging can reduce the HDL capacity in protecting lipoproteins against oxidation, and the probable mechanism is the decreasing PON1 activity of HDL [181]. Declined lipid efflux function may occur secondary to senescence-induced changes in cell morphology and energy metabolism, due to age-sensitive signaling pathways suppression, or maybe gene expression changes by a telomere position effect (TPE) of telomeric genes like ABCG1 [126]. It has been demonstrated that people with exceptional longevity and their offspring have larger HDL2-like particles and a lower prevalence of CVD [191,192].

The elevated fasting TG levels observed In older individuals may result from either increased triglyceride synthesis and VLDL production or a deficiency in lipoprotein lipase activity. LPL, also known as clearing factor lipase, is an enzyme dependent on insulin. The age-related rise in insulin resistance could contribute to changes in lipoprotein lipase activity [193], which can affect HDL contents and metabolism indirectly.

Regarding blood pressure, according to a study, a higher systolic blood pressure (SBP) level and age were associated with a lower HDL in both genders from ~20 to ~80 years of age [171]. The suggested mechanism for this relationship is that high concentrations of VLDL can stimulate the scavenger receptor B-I (SRB-I) in mitochondria, leading to heightened aldosterone production. The link between obesity, aldosterone, and hypertension may explain the mechanism behind lower HDL and increased BP with age. Consequently, reduced HDL levels might facilitate greater binding of VLDL or LDL to the SR–BI receptor, contributing to elevated BP.

**Figure 3:**
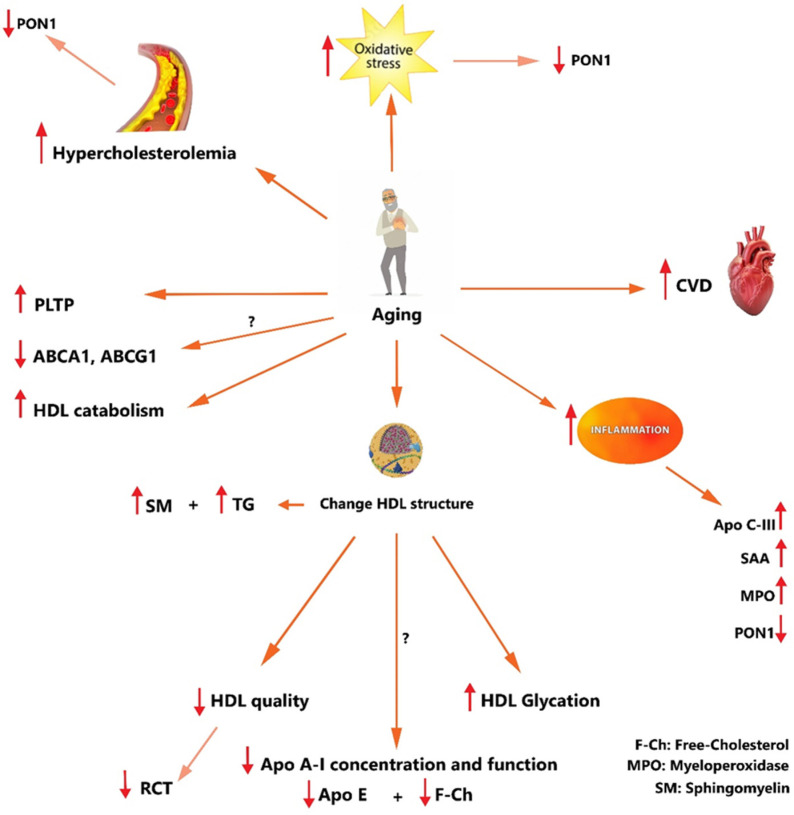
The interrelationships between aging, HDL, and CVD. ABCA1: ATP-binding cassette sub-family A member 1, ABCG1: ATP-binding cassette sub-family G member 1, Apo: Apolipoprotein, F-Ch: Free Cholesterol, HDL: High-Density Lipoprotein, MPO: Myeloperoxidase, PLTP: Phospholipid Transfer Protein, PON1: Paraoxonase 1, RCT: Reverse Cholesterol Transport, SAA: Serum Amyloid A, SM: Sphingomyelin, TG: Triglyceride.

In general, aging can cause multiple changes, like decreasing HDL levels and altering HDL particle size, composition, and function, which can reduce its cardioprotective effects. HDL may become less efficient at removing excess cholesterol from the bloodstream and protecting against CVDs with age. As people age, there is an increase in oxidative stress, which can damage HDL and reduce its function (Figure 3). 

## 8. Effect of Healthy Diet on HDL

Several studies have explored the impact of nutrient consumption on the structure and function of HDL. A review of clinical trials indicates that short-term intake of dietary antioxidants and alcohol is closely associated with enhanced HDL function in healthy individuals and those at cardiovascular risk, particularly in terms of CEC and HDL antioxidant properties [194]. Moreover, findings from Hernaez and co-workers suggest that the consumption of vegetables and a diet rich in antioxidants promotes HDL functions in humans. Furthermore, in individuals at cardiovascular risk, the intake of monounsaturated fatty acids (MUFAs) and long-chain polyunsaturated fatty acids (PUFAs) appears to affect HDL functions positively. Consistent findings reveal that long-chain PUFAs enhance HDL antioxidant capacity by boosting PON1 activity. Interestingly, MUFA interventions result in increased levels of CEC, CETP, and LCAT compared to saturated fatty acids (SFAs) and trans fatty acids (TFAs). Long-chain fatty acid intake also indicates improvements in CEC, CETP, and LCAT activities, especially when not compared to other unsaturated fatty acid interventions [194]. Changes in CEC could be caused by increments in apolipoprotein A-I [195]. Conversely, it has been suggested that higher doses of EPA and DHA (>3 g/day) may have an adverse impact on LCAT and HDL inflammatory indices while showing no alterations in CEC [194]. 

Saturated and trans fats exhibit contrasting effects compared to MUFAs and PUFAs, leading to lower CEC and LCAT and increased CETP. Dietary cholesterol intake, on the other hand, promotes increases in CEC, CETP, and LCAT activities, resulting in larger HDL particles and increased HDL diameter [196,197]. However, it is important to note that increases in CEC due to cholesterol intake may not necessarily indicate a better atheroprotective capacity of HDL [194]. Olive oil enriched with phenolic compounds, anthocyanins, carotene extracts, and supplements appears to have beneficial effects on HDL functionality. Meanwhile, the Mediterranean diet demonstrates a positive impact on various HDL functions, including CEC, antioxidant, anti-inflammatory, and endothelial protection capacities [198,199], while, interventions solely increasing dietary vegetable content have failed to improve the CETP, LCAT, and PON1 activities of HDL [200,201]. In a 2022 RCT, the well-formulated ketogenic diet (WFKD) was compared with the Mediterranean-plus diet (Med-Plus) in diabetes and pre-diabetes patients. The ketogenic diet demonstrated advantages, including a significant reduction in triglycerides and an increase in HDL cholesterol. However, it also led to an increase in LDL cholesterol, contrasting with the Med-Plus group where a reduction was observed [202]. Similar results were found in another study by Saslow et al. [203], while other studies found no difference between ketogenic diets and non-ketogenic diets in the case of HDL concentration [204].

The findings indicate the potential of dietary modifications to influence cardiovascular risk factors, especially HDL. However, further studies are required across diverse populations, various CVD statuses, and different age groups to enhance our understanding in this area. Significantly, it is essential to monitor not only HDL level but also its structure and function in diet intervention studies. Additionally, paying attention to changes in other biomolecules closely related to HDL is necessary.

## 9. Conclusions and Future Perspectives

CVD has become a prevalent chronic condition worldwide, and there is a growing need to deepen our understanding of its etiology and mechanism. Over the past century, there has been a substantial increase in global average life expectancy. However, the aging demographic is especially vulnerable to CVD, emerging as the leading cause of mortality among individuals aged 65 years and above. Moreover, in most populations, aging is often associated with factors such as weight gain, a sedentary lifestyle and lack of physical activity, insulin resistance, inappropriate eating habits, oxidative stress, and chronic inflammation, which can lead to various metabolic disorders. All mentioned factors can collectively contribute to metabolic disorders and compromise the efficacy of the lipoprotein plasma removal mechanism, leading to the development of CVDs. HDL, as an essential lipoprotein in lipid metabolism, is not an exception. Aging can affect various molecules, including proteins, lipids, RNAs, enzymes, receptors, and gene expressions. These changes can directly and indirectly modify the HDL structure. Additionally, the impact of aging on HDL is notable through oxidative stress and inflammation. The intensity of oxidative stress and inflammation in both elderly people and CVD patients can be affected by various factors, such as ethnicity, gender, genetics, lifestyle, diet, previous health conditions, etc. While significant progress has been made in understanding the structure and function of HDL in specific contexts, further studies are needed to explore HDL in various situations. This is essential for distinguishing different types of HDL and utilizing it as a potential predictive indicator for aging or cardiovascular disease (CVD). Moreover, additional research is needed to illustrate the exact mechanisms related to HDL itself and its relationship with other biomolecules, because HDL has not only cardioprotective effects but also antioxidant and roughly anti-inflammatory properties. 

In conclusion, it is vital to consider HDL function as an important biomarker for the prediction and evaluation of people at high risk of CVD or suffering from CVDs, specifically among the aged population. However, further studies are required to investigate the aging mechanisms through which cardiovascular health, particularly lipoprotein function, may be affected. This is crucial, as lipoprotein function and aging are linked to one another and exhibit overlapping mechanisms and features.
